# A Lightweight Vision Transformer and Retinal Biomarker Fusion Framework for Early Glaucoma Detection: Toward Improved Clinical Screening

**DOI:** 10.3390/jcm15145651

**Published:** 2026-07-18

**Authors:** Alifa Nasrin, Muhammad Bin Asif, Fatima Tuz Zahra, Afzal Haq Asif, Gausul Azam Khan, Md Arifuzzaman, Ramasamy Naidu, AKM Azad, Muhammad Ali Martuza

**Affiliations:** 1 Combined Military Hospital (CMH), Chattagram 4220, Bangladesh; 2 Clinical Department, Azerbaijani Medical University, Baku AZ1022, Azerbaijan; 3 Management Information System, College of Business Administration, King Faisal University, Al-Ahsa 31982, Saudi Arabia; 4 Pharmacy Practice, College of Clinical Pharmacy, King Faisal University, Al-Ahsa 31982, Saudi Arabia; 5 Department of Clinical Nutrition, College of Applied Medical Sciences, King Faisal University, Al-Ahsa 31982, Saudi Arabia; 6 Civil and Environmental Engineering, King Faisal University, Al-Ahsa 31982, Saudi Arabia; 7 Department of Computer Engineering, College of Computer Sciences & Information Technology, King Faisal University, Al-Ahsa 31982, Saudi Arabia; 8 Department of Mathematics and Statistics, Faculty of Science, Imam Mohammad Ibn Saud Islamic University (IMSIU), Riyadh 13318, Saudi Arabia; 9 Department of Computer Engineering, College of Computer, Qassim University, Buraydah 51452, Saudi Arabia

**Keywords:** glaucoma detection, retinal biomarkers, lightweight vision transformer, optic disc segmentation, deep learning (DL)

## Abstract

**Background/Objectives**: Glaucoma is a leading cause of irreversible blindness, and it is hard to catch early be-cause it rarely causes symptoms until real damage has already occurred. Existing au-tomated detection methods still miss the subtle structural changes that show up before vision loss begins. This study presents an automated framework based on optic nerve structure and retinal biomarkers, aimed at enabling earlier and more consistent glau-coma screening. The framework combines segmentation and classification in a single deep learning pipeline, trained and tested on the ORIGA and REFUGE2 fundus image datasets. Each image is resized, denoised with median filtering, and contrast-enhanced through histogram equalisation and normalisation, then cropped down to the optic nerve region using central cropping and intensity-based localisation. **Methods:** A custom encod-er–decoder CNN segments the optic disc and cup, and from that segmentation, we ex-tract biomarkers ophthalmologists already rely on, including the vertical cup-to-disc ratio and disc/cup area measurements. A Lightweight Vision Transformer separately learns broader structural patterns across the retina, and the two feature sets are fused and passed through a SoftMax classifier. **Results:** Segmentation accuracy was strong: Dice scores of 0.9082 for the optic disc and 0.9994 for the optic cup, IoU scores of 0.8351 and 0.9988, and an overall mean Dice of 0.9538 and mean IoU of 0.9169. Classification performance held up well, too, with high F1-score, recall, accuracy, and precision across normal and glaucomatous cases. **Conclusions:** The combination of interpretable, clinically established biomarkers with transformer-based global feature learning gives the framework the ability to support automated glaucoma risk assessment and demon-strates promising performance for glaucoma detection using retinal fundus images.

## 1. Introduction

Glaucoma is a progressive eye condition that causes damage to the optic nerve over a period of time. If not treated in a timely manner, it can cause irreversible vision loss [1]. In most cases, glaucoma occurs asymptomatically and it is challenging to diagnose it in its early stages [2]. Monitoring changes in the optic nerve and the retinal area is essential to identify individuals who are prone to the condition [3]. A boundary-aware transformer for joint optic disc and optic cup segmentation incorporates boundary information to enhance segmentation accuracy. The framework demonstrated improved delineation of retinal structures, supporting reliable glaucoma screening [4]. This ensures the preservation of vision. Evaluation of the retinal area is crucial for managing the condition [5]. Accurate screening also requires distinguishing primary glaucoma from secondary causes of elevated intraocular pressure, such as drug-induced uveitis, which can independently affect the optic nerve and complicate diagnosis.

The progressive optic nerve imaging and retinal biomarker framework is used for early detection of cases of glaucoma [6]. Various ophthalmic disorders, including inflammatory ocular diseases, require early diagnosis to prevent irreversible vision impairment, highlighting the importance of reliable computer-aided diagnostic systems [7]. This framework is based on the analysis of alterations taking place in the retina and optic nerve [8]. Emphasis is on identifying changes occurring in the optic cup and disc, as these changes are indicative of progression of disease [9]. The analysis of retinal biomarkers, such as cup-to-disc ratios and disc morphology, will help to identify early signs of glaucoma, as these changes may not be easily identified by routine examination [10]. UT-Net combines U-Net and transformer modules for simultaneous optic disc and optic cup segmentation and glaucoma detection [11]. The hybrid architecture effectively captured both local and global retinal features for improved diagnostic performance. Recent advances have also explored retina-inspired bioelectronic interfaces for intelligent visual perception and object recognition, further expanding the role of artificial intelligence in ophthalmic imaging applications [12].

Several existing techniques have also been used for glaucoma screening and include U-Net [13], VGG16 [14], InceptionV3 [15] and DenseNet121 [16]. U-Net’s encoder–decoder structure and skip connections make it very efficient for accurate optic cup and disc segmentation. It is sensitive to noise and prone to overfitting on smaller datasets. VGG16 is effective in feature extraction for classification, though it is computationally expensive and less effective for deeper feature extraction. InceptionV3 is effective in feature extraction, which is beneficial for improving accuracy in glaucoma screening. It is complex and makes training more time-consuming. DenseNet121 facilitates feature reuse and gradient flow, making it effective for segmentation and classification. This is computationally expensive and requires more memory.

The proposed framework for the early detection of glaucoma includes integration of progressive optic nerve analysis and the extraction of features from the retina. The process starts by preprocessing the images of the retina by resizing the images, removing noise from the images, enhancing the contrast of the images and normalising the images. The ROI of the images is automatically detected to ensure the images are concentrated around the optic nerve. A custom encoder–decoder CNN is then applied to segment the images into the OC and OD. Segmented images are used to extract the characteristics from the photos. Features are then classified into normal or glaucomatous cases.

### Objectives

- Develop an automated approach for the early detection and classification of glaucoma using structural changes in the optic nerve and retinal biomarkers.
- Utilise high-quality retinal fundus image datasets, specifically ORIGA and REFUGE2, to train and validate the proposed framework.
- Using a bespoke encoder–decoder CNN, precisely segment the OC and OD, and extract important retinal biomarkers, like a vertical cup-to-disc ratio, cup area, disc area, and border characteristics.
- Learn global image relationships using a lightweight Vision Transformer, fuse these features with extracted biomarkers and classify retinal images into normal or glaucomatous cases.

The study is structured in such a manner that Section 2 provides the literature survey and problem statement, Section 3 presents the proposed methodology, Section 4 presents the results and discussion, and Section 5 provides the conclusion to the study along with the future work that will be done.

## 2. Literature Survey

Alasmari et al. [17] suggested a transformer-based framework for glaucoma detection, using YOLOv11 for optic disc detection and MaskFormer with Swin Transformer for segmentation. The model used vCDR for classification and achieved strong segmentation performance but relied on a single biomarker. Islam et al. [18] developed a DL approach combining fundus and OCT images for glaucoma detection. Separate CNNs were used for each modality and the features were fused to improve accuracy. The results showed enhanced detection performance, but the framework did not include transformer models or progressive analysis. Bhattacharya et al. PY-Net, a dense pyramidal segmentation framework designed for accurate optic disc and optic cup localisation in retinal fundus images. The multi-scale feature extraction strategy improved segmentation performance across varying retinal structures [19]. Hasan et al. [20] presented an explainable Machine Learning (ML) framework for glaucoma detection using OCT images. The model used SHapley Additive exPlanations (SHAP) analysis and achieved high accuracy with AUC up to 1.00 for different stages. The approach relied on traditional machine learning and did not use transformer-based models. Zheng et al. [21] explain that to convolutional and transformer-based models, graph neural network architectures have emerged as powerful learning frameworks for a wide range of intelligent vision applications.

Prakash et al. [22] presented DB-SegNet, an optimised DL method for glaucoma detection that improves SegNet with multi-scale modules and a transformer, achieving high segmentation performance but lacking progressive and multi-biomarker analysis. Rekha et al. [23] presented a transformer–CNN-based method for optic disc and cup segmentation, improving feature representation but focusing only on segmentation without progressive or biomarker-based analysis. Chincholi et al. [24] explored transformer models for glaucoma diagnosis using retinal images. The study showed that transformers effectively capture global features and improve classification performance. It did not consider detailed retinal biomarkers or disease progression. Bowd et al. [25] introduced an autoencoder-based framework with Region of Interest (ROI) analysis for glaucoma detection in myopic eyes using OCT images. The approach achieved better performance through ROI-based reconstruction error compared to traditional methods. It was limited to OCT features and did not use transformer models or fusion techniques.

Zeppieri et al. [26] discussed Artificial Intelligence (AI)-based techniques for early glaucoma detection using various ML and DL methods. The study showed improved accuracy in detecting optic nerve changes. It provided a general overview and did not include transformer models or progressive biomarker analysis. Hernández et al. [27] introduced SpxViT, a superpixel-based Vision Transformer framework for glaucoma screening that improves model interpretability by integrating superpixel representations with transformer-based feature learning. Sharma et al. [28] presented a hybrid AI model for glaucoma detection using fundus images, improving classification performance but lacking transformer learning and progressive biomarker analysis. Chaglasian et al. [29] validated a glaucoma screening health score using OCT and clinical parameters like retinal nerve fibre layer thickness. The method effectively distinguished normal and glaucomatous eyes. It relied on predefined clinical features and did not use DL or transformer models. Wang et al. [30] presented a glaucoma detection method using hyperspectral imaging with a Vision Transformer, improving accuracy through spectral feature extraction but lacking progressive optic nerve and multi-biomarker analysis. Yurdakul et al. [31] introduced MaxGlaViT, a Vision Transformer-based method for early glaucoma stage detection, designed for efficient feature extraction and classification. Method accuracy in identifying various stages of glaucoma was encouraging. It focused mainly on classification and did not include retinal biomarker extraction or progressive optic nerve analysis. Table 1 presents a comparative analysis of current techniques.

### Problem Statement

Existing techniques for glaucoma detection have some limitations that make it hard for them to be used in real-world applications. Some of the techniques are complex, making them computationally expensive and time-consuming in training [17]. Some techniques, like the ones that use multi-modal [18] and OCT data, cannot be used in real-world applications since this data is not always available [32]. The transformer-based techniques require large data for training and cannot easily handle local features [23]. Some techniques cannot generalise well for various patient populations and some techniques cannot be easily trained and interpreted. Some techniques require data from clinical parameters and imaging data, which is costly, making it hard for them to be used in real-world applications. The proposed framework is efficient, accurate and effective in using fundus image data.

## 3. Proposed Methodology

**Figure 1:**
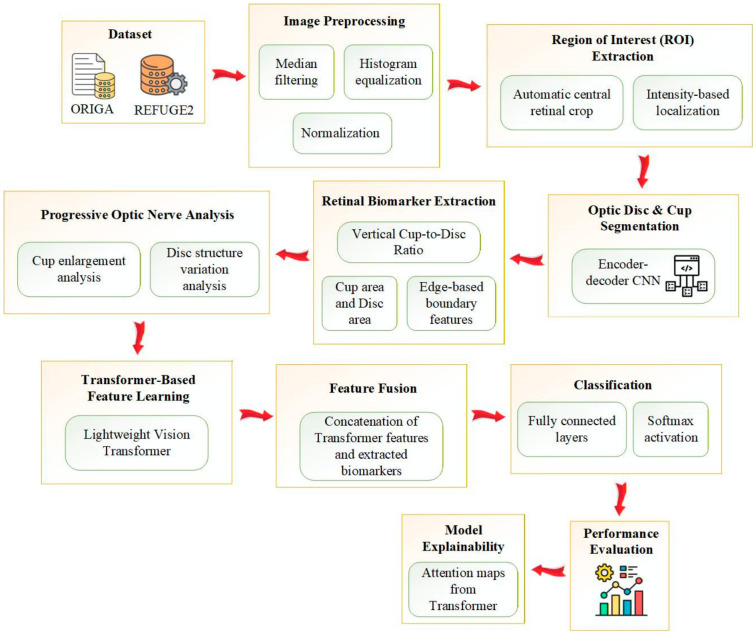
Overall schematic diagram of our proposed methodology.

The entire workflow of the proposed glaucoma screening framework is shown in Figure 1. It starts with the collection of retinal fundus images from the ORIGA and REFUGE2 databases. Preprocessing of the collected images is done using median filtering, histogram equalisation and image normalisation. The region of interest is extracted using central cropping and intensity-based localisation of the optic nerve area. OC and OD are segmented using a suggested encoder–decoder CNN. It facilitates the extraction of retinal biomarkers including the cup-to-disc ratio. Changes in the cup and disc structure are used to examine the optic nerve progression. A lightweight Vision Transformer is used for the extraction of global features and the fusion with the extracted biomarkers for the classification process. Performance evaluation and model explainability are done using standard metrics and attention maps. In summary, the key steps are: (1) image acquisition from ORIGA and REFUGE2; (2) preprocessing through resizing, median filtering, histogram equalisation, and normalisation; (3) ROI extraction via central cropping and intensity-based localisation; (4) OC and OD segmentation using the encoder–decoder CNN; (5) retinal biomarker extraction (vCDR, area, and border features); (6) global feature learning with the Lightweight Vision Transformer; (7) feature fusion and classification into normal or glaucomatous cases; and (8) performance evaluation and explainability analysis.

### 3.1. Dataset Description

Images of retinal fundus were obtained from the ORIGA and REFUGE2 datasets for the purpose of testing and training the suggested glaucoma screening method. The ORIGA dataset has 650 images with 520 for training and 130 for testing, whereas the REFUGE2 dataset has 1200 images for training and 240 for testing. The ORIGA and REFUGE2 datasets were partitioned prior to model development. For the ORIGA dataset, the official development set of 520 images was divided into 416 training images (80%) and 104 validation images (20%), while the official independent test set of 130 images was reserved exclusively for final performance evaluation. Similarly, the REFUGE2 development set of 1200 images was split into 960 training images (80%) and 240 validation images (20%), whereas the official independent test set of 240 images was retained solely for testing. Subject/eye-disjoint partitioning was strictly maintained to ensure that images from the same subject or eye did not appear in more than one subset. All preprocessing operations were performed after dataset partitioning, and the independent test sets were never used during model training, hyperparameter tuning, or model selection. These measures eliminated data leakage and ensured that the reported segmentation and classification performance was evaluated on previously unseen retinal fundus images.

#### 3.1.1. ORIGA Dataset

The Optic Nerve Head Image Grading and Analysis (ORIGA) dataset [33] is a public domain fundus image dataset specifically designed for the task of glaucoma detection. It consists of maximum-resolution colour images of the retina which are annotated with the presence or absence of glaucoma, as well as information relating to the optic disc. The dataset can be used for classification and segmentation allowing the analysis of structures within the optic nerve head. It is often utilised for the development and evaluation of automatic glaucoma screening systems.

#### 3.1.2. REFUGE2 Dataset

REFUGE2 [34] is a large-scale retinal fundus image dataset designed for the Retinal Fundus Glaucoma Challenge. It is used for glaucoma detection and segmentation. The dataset has around 2000 images with detailed annotations. It includes glaucoma information, optic disc and cup segmentation and fovea localisation. It has images from multiple devices and qualities, making it a practical dataset. It is used for various applications such as image segmentation and classification. It serves as a common criterion for assessing how well automated glaucoma detection systems function.

### 3.2. Image Preprocessing

Preprocessing pictures is an essential step in improving the quality of retinal fundus photographs. The quality of images is improved through image preprocessing, which ultimately boosts the effectiveness of image segmentation and classification algorithms. Within the context of this study, image preprocessing entails resizing, median filtering, histogram equalisation, and normalisation of the images to ensure that they are ready for feature extraction.

#### 3.2.1. Image Resizing

Image resizing is done to transform all the retinal fundus images into a standard size, and this operation is performed to achieve uniformity in the size of the images, which are further used as input to the DL models. This operation reduces the complexity and makes it computationally efficient to perform batch processing on the images, maintaining the structure while resizing the images to the desired size. Equation (1) indicates the image resizing:

(1) $$I^{\prime }\left(x,y\right)=I\left(x\cdot \frac{W}{W^{\prime }},y\cdot \frac{H}{H^{\prime }}\right)$$

where I denotes the original image, $I^{\prime }$ denotes the resized image, W,H denotes the original width and height and $W^{\prime },H^{\prime }$ denotes the target dimensions.

#### 3.2.2. Median Filtering

Median filtering is used for noise removal from retinal images without affecting important edge information. The procedure entails substituting the median value of nearby pixels for each pixel. This filtering is effective against salt and pepper noise. The method ensures that fine details, such as boundaries of the optic disc, are not distorted. This enhances image clarity for retinal features, which is important for accurate biomarker detection and segmentation in glaucoma analysis. Beyond classical filtering, deep learning-based denoising and enhancement approaches have also been explored in other medical imaging contexts, including super-resolution ultrasound micro vessels, imaging [35], denoising filtering in ultrasound localisation microscopy [36], generative adversarial networks with pyramid coordinate attention for robust image denoising [37,38] and gated Swin transformer-based image denoising [39], suggesting potential directions for further enhancing retinal image preprocessing in future work. Equation (2) indicates median filtering:

(2) $$I^{\prime }\left(x,y\right)=\mathrm{median}\left\{I\left(i,j\right)|\left(i,j\right)\in N\left(x,y\right)\right\}$$

where N(x,y) represents the neighbourhood window around pixel (x,y).

#### 3.2.3. Histogram Equalisation

Histogram equalisation is a method for enhancing retinal picture contrast. It does this by equalising the intensity values of pixels. It increases the brightness of dark areas of the image. It is useful for enhancing the visibility of important features of the image such as the OC and OD. It is used to improve the visibility of small changes in features of the image which may not otherwise be clearly visible. It improves the image quality and this increases the efficiency of feature extraction. Equation (3) indicates histogram equalisation:

(3) $$I^{\prime }\left(x,y\right)=\;\mathrm{round}\;\left(\left(L-1\right)\cdot \sum_{k=0}^{I\left(x,y\right)}p\left(k\right)\right)$$

where L represents the number of intensity levels and p(k) represents the probability distribution of intensity level k.

#### 3.2.4. Normalisation

Normalising the image ensures that the pixel intensity is on a standard scale, usually ranging from 0 to 1. This helps in minimising the effect of the environment in which the image was taken. Normalisation is used to stabilise the learning of DL models. It helps in speeding up the convergence of the learning process. Normalisation ensures that the features learned from the image are reliable and comparable with other images. Equation (4) indicates normalisation:

(4) $$I^{\prime }\left(x,y\right)=\frac{I\left(x,y\right)-I_{\min}}{I_{\max}-I_{\min}}$$

where $I_{\min\;},\;I_{\max\;}$ represent minimum and maximum pixel values.

### 3.3. Region-of-Interest Extraction

Region-of-interest extraction is concerned with extracting the most important part of the retinal picture, particularly the area around the optic nerve head. ROI is important for glaucoma analysis. The ROI is extracted to avoid unnecessary information in the image. By focusing only on important features of the image, ROI extraction is useful for improving the accuracy of image segmentation. In image analysis, ROI is extracted through central retinal cropping and intensity-based localisation.

#### 3.3.1. Central Retinal Cropping

Central retinal cropping is a technique used to crop the middle section of fundus image where the OD is generally found. This cropping method eliminates unnecessary background areas, including dark borders and peripheral areas. It is useful for maintaining clinically useful information within the image. By reducing the image size and focusing on the key area of the retina, this cropping method improves the efficiency of the next processing stage. Equation (5) indicates central retinal cropping:

(5) $$I_{ROI}\left(x,y\right)=I\left(x+x_{c},y+y_{c}\right)$$

where $\left(x_{c},y_{c}\right)$ represent centre coordinates of the image and $I_{\mathrm{ROI}}$ represents the cropped region of interest.

#### 3.3.2. Intensity-Based Localisation

In intensity-based localisation, the optic disc region is localised based on the increased level of brightness in comparison to the neighbouring regions in the retina. This technique involves the use of thresholding methods to detect the regions with a high level of pixel intensity which corresponds to the optic nerve head. This technique is effective for accurately localising ROI, even in the presence of varying illumination conditions. It accurately highlights the optic disc region, thus enhancing the reliability of the segmentation process. Equation (6) indicates intensity-based localisation:

(6) $$I_{ROI}\left(x,y\right)=\{\begin{array}{ll} 1, & \;\mathrm{if}\;I\left(x,y\right)>T\\ 0, & \;\mathrm{otherwise}\; \end{array}$$

where T represents intensity threshold and I(x,y) represents pixel intensity.

### 3.4. Optic Disc and Cup Segmentation Using Encoder–Decoder CNN

Segmentation of the OC and OD areas is done with the help of a custom encoder–decoder convolutional neural network. This neural network helps in the precise segmentation of the areas. The encoder part of the neural network focuses on the features from the image, while the decoder part focuses on the reconstruction of the features. This procedure aids in the accurate division of the OD and OC regions, which are important for glaucoma. The process of biomarker extraction plays an important part in the early classification of glaucoma.

**Figure 2:**
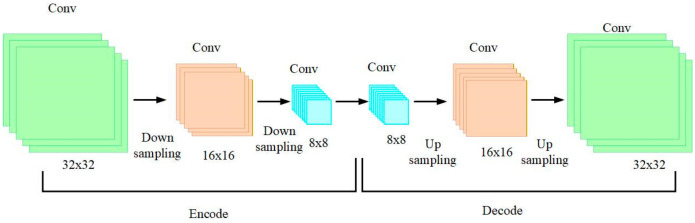
Encoder–decoder CNN architecture used in our study.

The proposed encoder–decoder CNN consists of four encoder blocks and four corresponding decoder blocks. Each encoder block contains two convolutional layers with a kernel size of 3 × 3, followed by Batch Normalisation and ReLU activation to improve feature learning and training stability. A 2 × 2 max-pooling layer is employed after each encoder block for spatial downsampling. The decoder reconstructs the segmentation maps using 2 × 2 transposed convolutions for upsampling, followed by convolutional layers with Batch Normalisation and ReLU activation. Skip connections are established between corresponding encoder and decoder blocks to preserve fine spatial details and improve boundary localisation of the optic disc and optic cup. The final segmentation layer applies a 1 × 1 convolution followed by Softmax activation to generate pixel-wise probability maps for optic disc and optic cup segmentation. The Convolution Operation is shown in Equation (7):

(7) $$F\left(x,y\right)=\sum_{i}\sum_{j}I\left(x+i,y+j\right)\cdot K\left(i,j\right)$$

where F(x,y) represents the outcome feature map value at pixel location (x,y), I(x+i,y+j) represents input image pixel values in the neighbourhood around (x,y), K(i,j) represents Kernel (filter) values used to extract features, i,j are indices representing the size of the kernel, and Σ is the summation operation over the kernel region. Segmentation output Equation (8):

(8) $$S\left(x,y\right)=\mathrm{argmax}P_{c}\left(x,y\right)$$

where S(x,y) represents the final predicted class label at pixel (x,y), $P_{c}\left(x,y\right)$ represents the probability of class c at pixel (x,y), *c* represents the class index (e.g., background, OD, OC) and argmax represents the function that selects the class with the highest probability. Figure 2 shows the encoder–decoder CNN.

### 3.5. Retinal Biomarker Extraction

In retinal biomarker extraction, emphasis is placed on extracting significant structural information from the extracted optic disc and cup areas. Biomarkers play a significant role in identifying changes in structures that are linked with glaucoma. Quantification of changes in size, shape and boundaries is an important aspect of extracting significant clinical biomarkers for detecting glaucoma. Significant biomarkers extracted are vCDR, OC Area and OD Area and Edge-Based Boundaries. These biomarkers were selected because they correspond directly to structural criteria routinely evaluated during clinical optic nerve head examination, allowing the proposed framework to generate predictions that remain clinically interpretable rather than relying solely on learned deep features. The extracted biomarkers, including the vertical cup-to-disc ratio (vCDR), optic cup area, optic disc area, and edge-based boundary characteristics, provide quantitative measurements of structural changes associated with glaucomatous optic neuropathy. Their integration with transformer-based image features enables the framework to combine clinically meaningful structural information with high-level visual representations for more reliable glaucoma detection.

#### 3.5.1. Vertical Cup-to-Disc Ratio

Vertical cup-to-disc ratio is an important biomarker for diagnosing glaucoma by measuring the ratio of the vertical diameters of OC and OD. An increase in OC to OD ratio is an indication of an increase in cup size, a common finding in glaucoma patients. Measurement is useful for detecting damage to the optic nerve. OC to OD ratio is a widely accepted method for diagnosing glaucoma, as it is simple, relevant and effective for differentiating between glaucomatous and non-glaucomatous eyes. Equation (9) indicates the vertical OC to OD ratio:

(9) $$vCDR=\frac{D_{\mathrm{cup}\;}}{D_{\mathrm{disc}\;}}$$

where $D_{\mathrm{cup}\;}$ represents the vertical diameter of OC and $D_{\mathrm{disc}\;}$ represents the vertical diameter of OD. Clinically, the vertical cup-to-disc ratio is one of the most widely accepted structural biomarkers for glaucoma assessment because progressive enlargement of the optic cup relative to the optic disc reflects loss of neuroretinal rim tissue and retinal ganglion cells. Larger vCDR values are generally considered more suggestive of glaucomatous optic nerve damage; however, the measurement should always be interpreted together with optic disc size and morphology, since physiological variations in disc size may influence the apparent cup size. In addition, noticeable inter-eye asymmetry in vCDR is recognised as an important clinical indicator that may suggest the early development of glaucomatous optic neuropathy. Therefore, the proposed framework combines vCDR with additional structural biomarkers to improve the reliability and clinical interpretability of glaucoma detection.

#### 3.5.2. Cup Area and Disc Area

The OC area and OD area indicate sizes of OC and OD areas, respectively, after segmentation. These values carry significant information regarding changes in the structure of the retina. During a case of glaucoma, the cup area is seen to increase in relation to the disc area, reflecting damage to the optic nerve. Calculating OC and OD areas is useful for assessing the severity of the disease and for making a reliable analysis. Equation (10) indicates the cup area and disc area:

(10) $$A=\sum_{x,y}S\left(x,y\right)$$

where *A* represents area (cup or disc) and S(x,y) represents segmented region pixels. The optic disc area has independent clinical significance because physiological differences in disc size directly influence the interpretation of optic cup enlargement. Eyes with relatively larger optic discs generally exhibit proportionally larger physiological cups, whereas smaller optic discs may conceal early glaucomatous changes. Consequently, evaluating the optic cup area together with the optic disc area provides a more reliable structural assessment than considering either measurement alone. Progressive enlargement of the cup area relative to the disc area reflects neuroretinal rim loss associated with retinal ganglion cell degeneration and is therefore regarded as an important structural biomarker for glaucoma screening and longitudinal disease monitoring.

#### 3.5.3. Edge-Based Boundary Features

The edge-based features for boundary characteristics concentrate on the identification of OC and OD borders, which can be achieved by detecting the variation in the image intensity. These features can be useful in the identification of the shape and boundary characteristics of the structures, which can be significant for precise segmentation. Irregularities or distortions in the boundaries can also be indicative of the occurrence of glaucoma. The precision of the assessment can be improved through the differentiation of the edges. Equation (11) indicates edge-based features:

(11) $$G=\sqrt{{\left(\frac{\partial I}{\partial x}\right)}^{2}+{\left(\frac{\partial I}{\partial y}\right)}^{2}}$$

where G represents edge strength and $\frac{\partial I}{\partial x},\frac{\partial I}{\partial y}$ represent image gradients in *x* and *y* directions. Edge-based boundary features provide a computational representation of the neuroretinal rim contour, as assessed routinely during clinical fundus examination. Clinically, neuroretinal rim integrity is commonly evaluated using the ISNT rule, which states that, in healthy eyes, the neuroretinal rim thickness generally follows the order Inferior > Superior > Nasal > Temporal (I > S > N > T). Deviations from this pattern, together with focal rim thinning, notching, or irregular optic disc and optic cup contours, are recognised structural indicators of glaucomatous optic neuropathy. In the proposed framework, the intensity-gradient-based edge strength, as described in Equation (11), serves as a computational indicator of boundary sharpness and irregularity. Abrupt or asymmetric gradient variations along the optic disc and optic cup boundaries may indicate structural deformation associated with neuroretinal rim thinning, thereby providing clinically meaningful information that complements conventional retinal biomarkers such as the vertical cup-to-disc ratio and optic nerve head area measurements.

### 3.6. Progressive Optic Nerve Analyses

Progressive optic nerve analysis is concerned with identifying changes in the OD and OC. The changes are important for detecting early stages of glaucoma, since it affects the optic nerve structure gradually. By identifying changes in size and structure, it is possible to detect abnormalities at an initial stage. Efficient computational frameworks have been widely investigated to improve scalability and computational performance in large-scale intelligent systems [40]. The main approaches used in progressive optic nerve analysis are cup enlargement analysis and disc structure variation analysis. Recent advances in efficient data representation and high-performance computing have contributed to accelerating large-scale intelligent data processing [41].

#### 3.6.1. Cup Enlargement Analyses

Measuring variations in optic cup size relative to the optic disc is known as cup enlargement analysis. The loss of nerve fibres in glaucoma causes the optic cup to enlarge. This is an important aspect of glaucoma detection. Changes in cup size are used to detect abnormalities at an early stage. This is a useful method for detecting abnormalities at early stages by monitoring changes in cup size at different stages. Equation (12) indicates cup enlargement analysis:

(12) $$\Delta C=A_{\mathrm{cup}\;}^{t2}-A_{\mathrm{cup}\;}^{t1}$$

where ΔC represents change in cup area and $A_{\mathrm{cup}\;}^{t1},A_{\mathrm{cup}\;}^{t2}$ represent cup area at two different stages.

#### 3.6.2. Disc Structure Variation Analysis

In disc structure variation analysis, the shape, size and boundaries of the od are analysed. Any deformation or abnormality in the disc structure may be an indicator of the damage to the optic nerve which occurs in glaucoma. Process of disc structure variation analysis enables a deeper understanding of the influence of glaucoma on the overall disc structure of the optic disc. It becomes easier to differentiate between normal and abnormal situations through disc structure variation analysis. Equation (13) indicates disc structure variation analysis:

(13) $$D_{var}=\|S_{disc}^{t2}-S_{disc}^{t1}\|$$

where $D_{\mathrm{var}\;}$ represents disc variation measure, $S_{disc}^{t1},S_{disc}^{t2}$ represent disc structures at different stages and ‖ − ‖ represents the difference or distance measure.

### 3.7. Transformer-Based Feature Learning Using Lightweight Vision Transformer

Transformer-based feature learning employs a Lightweight Vision Transformer to efficiently capture global relationships and contextual information from the retinal images, which is vital in the accurate detection of glaucoma. The entire image is split into smaller patches, and each patch is processed to effectively learn meaningful representations. This helps the model understand the long-range dependencies between different regions of the retina. This is a lightweight solution which reduces the complexity and makes the solution more efficient in the detection and classification, as it incorporates both local and global information. Equation (14) indicates Lightweight Vision Transformer:

(14) $$z_{0}=\left[x_{\mathrm{class}};x_{p}^{1}E;x_{p}^{2}E;\ldots ;x_{p}^{N}E\right]+E_{\mathrm{pos}\;}$$

where $z_{0}$ represents the input sequence to the transformer encoder, $x_{\mathrm{class}:}$ represents the learnable classification token representing the whole image, $x_{p}^{1},x_{p}^{2},\ldots ,x_{p}^{N}$ represent image patches obtained by dividing the image, *E* represents the linear projection matrix, N represents the number of image patches, $E_{\mathrm{pos}}$ represents the positional encoding added to retain spatial information and [ ; ] represents the concatenation operation.

(15) $$\mathrm{Attention}\left(Q,K,V\right)=\mathrm{Softmax}\left(\frac{QK^{T}}{\sqrt{d_{k}}}\right)V$$

where Q (Query) denotes current patch features, K (Key) denotes all patch features for comparison, V (Value) contains the actual information to be aggregated, $K^{T}$ represents transpose of the key matrix, $d_{k}$ represents the dimension of key vectors, $\sqrt{d_{k}}$ represents the scaling factor for gradient stabilisation and Softmax function converts values into probability distribution. The Lightweight Vision Transformer processes retinal fundus images resized to 224 × 224 pixels, which are divided into 8 × 8 non-overlapping image patches. Each patch is projected into a learnable embedding space and combined with positional embeddings before being passed through four to six transformer encoder layers. Each encoder layer consists of multi-head self-attention, Layer Normalisation, residual connections, and a feed-forward multilayer perceptron with GELU activation. The transformer learns global contextual relationships among retinal structures while maintaining computational efficiency. The final transformer feature representation is concatenated with the extracted retinal biomarkers to improve glaucoma classification performance.

### 3.8. Feature Fusion Using Concatenation

Feature fusion uses combined features from different sources to improve glaucoma detection precision. Features from the Lightweight Vision Transformer are fused with other clinically significant features from the retina, such as vCDR, cup area and disc area. This fusion of features can be done using the concatenation method, where the features from the two sources are combined. This improves the accuracy of the categorisation and the detection of the phases of glaucoma by enabling the model to fully comprehend the features from the retina. Equation (16) indicates concatenation:

(16) $$F_{\mathrm{fused}}=\left[F_{\mathrm{transformer}};F_{\mathrm{biomarkers}}\right]$$

where $F_{\mathrm{fused}}$ represents the final combined feature vector, $F_{\mathrm{transformer}}$ represents the features extracted from the Lightweight Vision Transformer, $F_{\mathrm{biomarkers}}$ represents the extracted retinal biomarkers (vCDR, cup area, disc area, etc.) and [;] represents concatenation operation.

### 3.9. Classification

The final stage of the framework is classification, in which the combined features are used to identify glaucoma in the retinal image. This stage transforms the extracted and combined features into useful class labels. This ensures the precise classification of images for different stages of glaucoma. The classification in this framework is performed by fully connected layers and softmax activation.

#### 3.9.1. Fully Connected Layers

The aim of classification is to employ fully connected layers to discover intricate correlations between the extracted and fused characteristics. In this layer, every neuron is connected to all input features. It is used to transform features into class scores using weights and biases. It helps map the learned feature representations into a decision space, enabling the model to differentiate between normal and glaucomatous cases. The fused feature vector obtained from the Lightweight Vision Transformer and retinal biomarkers is passed through fully connected layers for classification. ReLU activation is applied after each fully connected layer to introduce non-linearity, while the final output layer employs Softmax activation to generate class probabilities corresponding to the normal and glaucoma categories. It is used to refine feature importance. It is used to reduce dimensional complexity. Equation (17) indicates fully connected layers:

(17) z=W⋅x+b

where z represents the output vector, *W* represents the weight matrix, x represents the initial feature vector, and b represents the bias term.

#### 3.9.2. Softmax Activation

The softmax activation function is used to transform the output scores of the fully connected layer into probability scores. This guarantees that the probability sum will always equal one. Multi-class classification problems employ it. A value that indicates the likelihood of the initial belonging to a class is the output of the softmax activation function. The class with the highest probability is selected as the method outcome. This is used to make decisions in a clear and interpretable manner for glaucoma classification. It improves the interpretability of the decisions made by the model. It ensures a normalised probability distribution over the classes. Equation (18) indicates softmax activation:

(18) $$P\left(y_{i}\right)=\frac{e^{z_{i}}}{\sum_{j=1}^{C}e^{z_{j}}}$$

where $P\left(y_{i}\right)$ represents the probability of class i, $z_{i}$ represents the outcome score for class i and C represents the total values of classes.

### 3.10. Model Explainability

Explainability of the model is another key feature of the suggested framework. Explainability is a critical aspect of the proposed framework because it guarantees that the process of determining decisions is transparent. In the proposed framework, the attention maps created using the Lightweight Vision regions of retinal vision that the method uses most frequently for predictions are shown using transformers. These maps highlight the important areas of the image, such as the OD and the OC, ensuring that the model is using relevant areas of the image in its predictions. Algorithm 1 shows the Lightweight Vision Transformer for glaucoma classification.
**Algorithm 1:** Lightweight Vision Transformer for Glaucoma Classification**Input:** Retinal Image I **Output:** Class Label (Normal/Glaucoma) Step 1: Preprocessing Resize image I to fixed size Normalize pixel values Step 2: Patch Extraction Divide image I into N patches: {P1, P2, …, PN} Step 3: Patch Embedding For each patch Pi: Flatten Pi Map to embedding vector using linear projection End For Step 4: Add Positional Encoding Add positional embeddings to each patch embedding Step 5: Append Class Token Add learnable class token to sequence Step 6: Transformer Encoder For each encoder layer L: Apply Multi-Head Self-Attention: Attention (Q, K, V) Apply Feed Forward Network:
FFN(x)=max(0,xW1+b1)W2+b2
Apply Layer Normalization End For Step 7: Feature Extraction Extract final representation from class token Step 8: Classification Pass features through Fully Connected Layer Apply Softmax: P(y)=exp(z)/Σexp(z)
Step 9: Output Return class with highest probability

## 4. Results and Discussion

The suggested framework ensures that it performs well and consistently in detecting glaucoma based on the integration and combination of preprocessing, segmentation, feature learning and biomarker extraction. The method ensures dependable categorisation for both normal and glaucoma patients by performing well in terms of precision, accuracy, recall, and F1-score. The proposed model ensures accurate segmentation based on the high values obtained for the Dice score, thus ensuring accurate segmentation for the OC and OD. Combination of retinal biomarkers and Lightweight Vision Transformer features ensures better detection for glaucoma patients. The attention mechanism ensures better explainability for the proposed model based on important retinal features, as shown in Table 2.

Hyperparameter Details

The suggested framework is based on a custom CNN encoder–decoder with deep supervision for precise optic disc (OD) and optic cup (OC) segmentation, consisting of four encoder blocks and four decoder blocks. Each encoder block contains two 3 × 3 convolutional layers, followed by Batch Normalisation and ReLU activation, while 2 × 2 max-pooling is used for downsampling. The decoder employs 2 × 2 transposed convolutions for upsampling together with skip connections to recover spatial information. The final segmentation layer uses a 1 × 1 convolution with Softmax activation, and the network is optimised using a combined Dice loss and Cross-Entropy loss. Preprocessing operations include image resizing, median filtering, CLAHE, normalisation, and ROI extraction. Retinal biomarkers, including the vertical cup-to-disc ratio (vCDR), cup area, disc area, and diameter measurements, are extracted and fused with global image features learned by a Lightweight Vision Transformer using 8 × 8 image patches, four to six transformer encoder layers, multi-head self-attention, Layer Normalisation, and GELU activation. The fused features are classified through fully connected layers using Softmax activation. Model optimisation is performed using the Adam optimiser with a batch size of 16 and a learning rate of 0.0001.

### 4.1. Performance Evaluation Metrics

Accuracy: Accuracy of the model is determined by dividing the number of correctly predicted samples by the total number of samples. Performance matrix IoU, F1-score, dice score, recall, accuracy and precision are shown by Equations (18)–(24).

(19) $$\mathrm{Accuracy}\;=\frac{TP+TN}{TP+TN+FP+FN}$$

Precision: The number of accurately anticipated positive samples is a measure of precision. Precision is important in case false positives need to be minimised.

(20) $$\;\mathrm{Precision}\;=\frac{TP}{TP+FP}$$

Recall: The value of real favourable cases that the method accurately detected is called recall. It is a key measure when missing positive cases is costly.

(21) $$\mathrm{Recall}=\frac{TP}{TP+FN}$$

F1-score: The F1-score is the harmonic mean of recall and precision. It is advantageous when both false positives and false negatives are substantial.

(22) $$F1\text{-}\mathrm{score}=2\times \frac{\;\mathrm{Precision}\;\times \;\mathrm{Recall}\;}{\;\mathrm{Precision}\;+\;\mathrm{Recall}\;}$$

Dice score: The segmentation of the ground truth and the prediction is compared using the Dice score. It is employed to gauge the precision of OC and OD segmentation.

(23) $$\mathrm{Dice}=\frac{2\left|X\cap Y\right|}{\left|X\right|+\left|Y\right|}$$

IoU: The extent to which the ground truth and anticipated regions are assessed using IoU. IoU reflects the segmentation accuracy.

(24) $$\mathrm{IoU}=\frac{\left|X\cap Y\right|}{\left|X\cup Y\right|}$$

where *FP* stands for False Positive, *FN* for False Negative, *TP* for True Positive, and *TN* for True Negative, X represents predicted region and Y represents ground truth region.

### 4.2. Confusion Matrix Analysis

**Figure 3:**
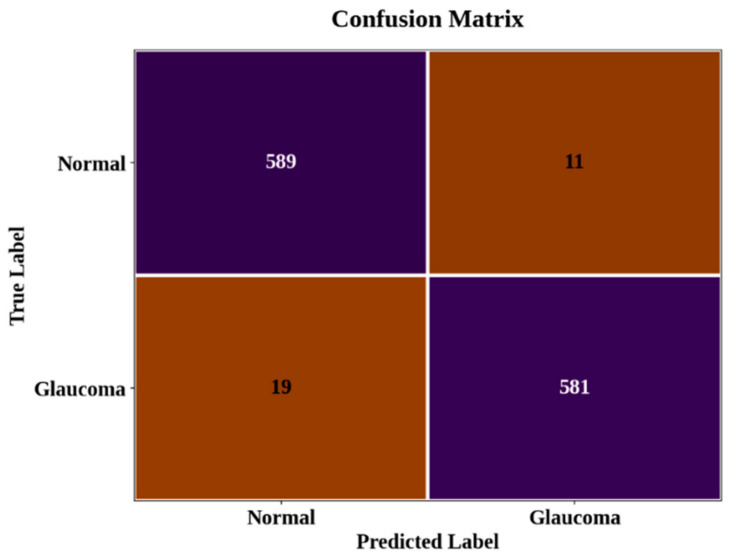
Confusion matrix.

The method performance is displayed in the confusion matrix separating class of normal eyes from the class of glaucoma patients in Figure 3. Out of 600, the model correctly identified 589 as normal, while only 11 were incorrectly identified as glaucoma patients. Out of 600 glaucoma patients, the model correctly identified 581 while only 19 were incorrectly identified as normal patients. The maximum accuracy and reliability of the method is highly sensitive and specific for glaucoma detection, as shown by the low number of incorrect identifications.

### 4.3. Accuracy and Loss Analyses

**Figure 4:**
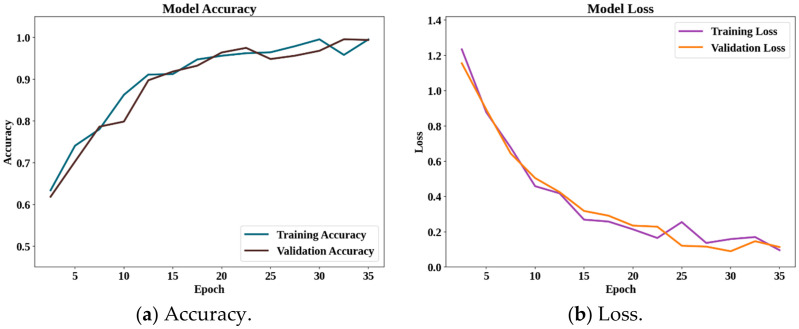
Accuracy and loss.

Figure 4 shows the model accuracy and losses for 35 epochs. The training accuracy increases from 0.75 to near 0.99 and validation accuracy increases from 0.73 to 0.97, showing excellent generalisation capability. At the same time, training losses decrease from 0.55 to 0.02 and validation losses decrease from 0.60 to 0.04, showing fewer errors. It is clear that the method is learning effectively, exhibiting excellent accuracy, minimal losses, stability and robustness throughout.

### 4.4. Original and Preprocessing Image Glaucoma and Non-Glaucoma Analysis

**Figure 5:**
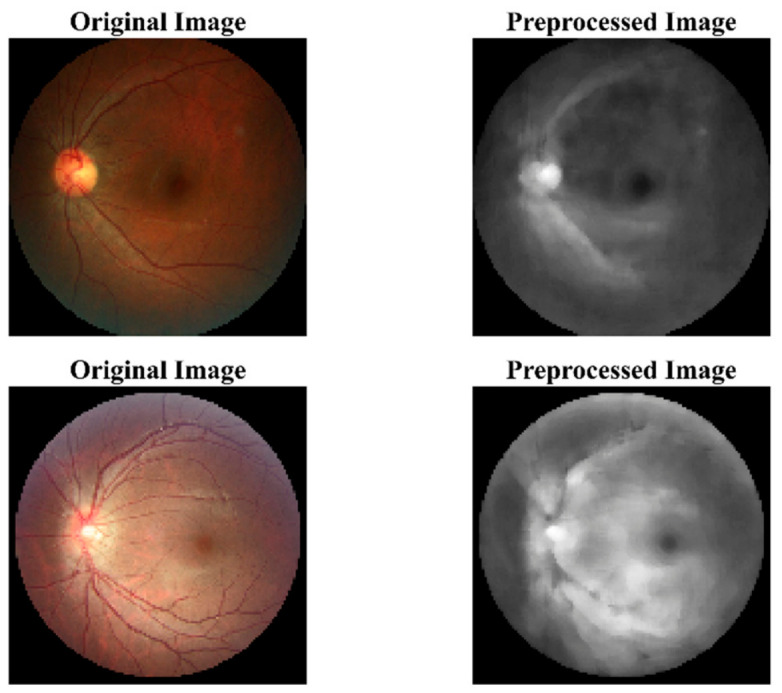
Original and preprocessing image glaucoma and non-glaucoma (source fundus images from ORIGA [28] and REFUGE2 [29] datasets).

The original retinal fundus images of the patients with and without glaucoma and the corresponding preprocessed retinal images are shown in Figure 5. In the original-coloured retinal scan images, the optic disc and blood vessel details are prominent and the preprocessing of the retinal scan images converts them to grayscale, thereby highlighting the important anatomical details and removing noise. This helps in the clear visualisation of the important details of the retinal scan pictures, increasing the precision of the model in distinguishing between glaucoma and non-glaucoma patients.

### 4.5. Original and Glaucoma Prediction Image Analysis

**Figure 6:**
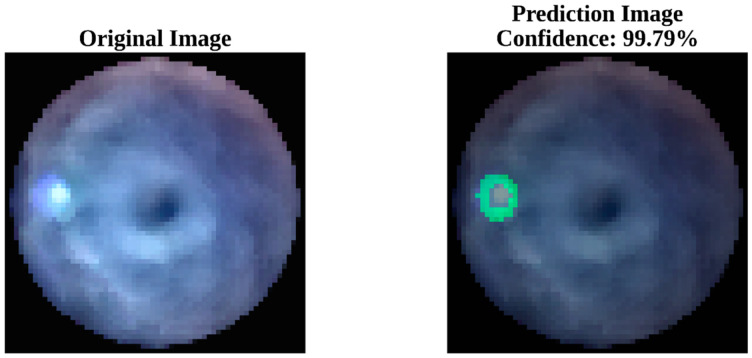
Original and glaucoma prediction images (source fundus image from REFUGE2 [29] dataset; prediction overlay generated by the proposed model).

The original retinal image and its glaucoma prediction output are shown in Figure 6. The original image displays the anatomical structure while the prediction shows a specific region with a confidence score of 99.79%. The green-marked region shows the detection of glaucomatous features by the model which can be used to classify the regions of the disease with high confidence. The high confidence level of the model shows its robustness, which can be used for the detection of glaucoma with maximum accuracy.

### 4.6. Overall Dice and IoU Performance Analysis

**Figure 7:**
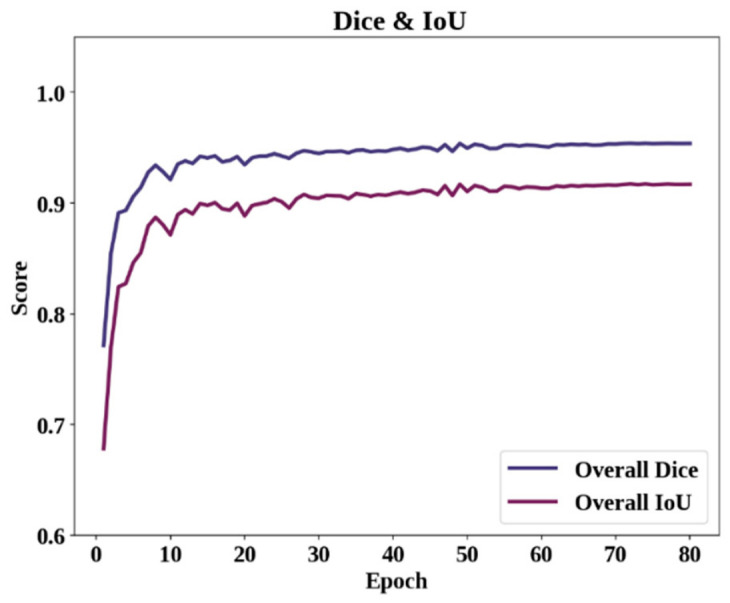
Overall Dice and IoU performance.

The performance of the model in segmenting images using the Dice and IoU scores for 80 epochs is shown in Figure 7. It is evident that the method performance improves steadily for both Dice and IoU scores. The scores start from around 0.65 and increase steadily to reach a constant state around 0.95 for Dice and 0.93 for IoU. This demonstrates the learning and improvement in performance of the method. The constant state of curves also shows that the model has successfully segmented the images by achieving a high overlap between the segmented and actual images. The proposed framework achieved a Dice score of 0.9994 for optic cup segmentation, the reported performance was obtained using independent test sets that remained completely isolated from the training and validation stages. The strict subject/eye-disjoint partitioning strategy and the exclusive use of the official test sets for final evaluation prevented data leakage between the training and testing datasets. Therefore, the reported segmentation accuracy reflects the model’s generalisation capability in previously unseen retinal fundus images within the ORIGA and REFUGE2 benchmark datasets.

The segmentation performance of the suggested method for OD and OC is shown in Table 3. With an IoU of 0.8351 and a dice score of 0.9082, OD successfully segmented the disc region. The OC shows very high performance with a Dice score of 0.9994 and IoU of 0.9988, reflecting near-perfect segmentation. The overall mean values of 0.9538 Dice and 0.9169 IoU demonstrate that the model provides highly precise and consistent segmentation results across both regions.

### 4.7. ROC Curve Analyses

**Figure 8:**
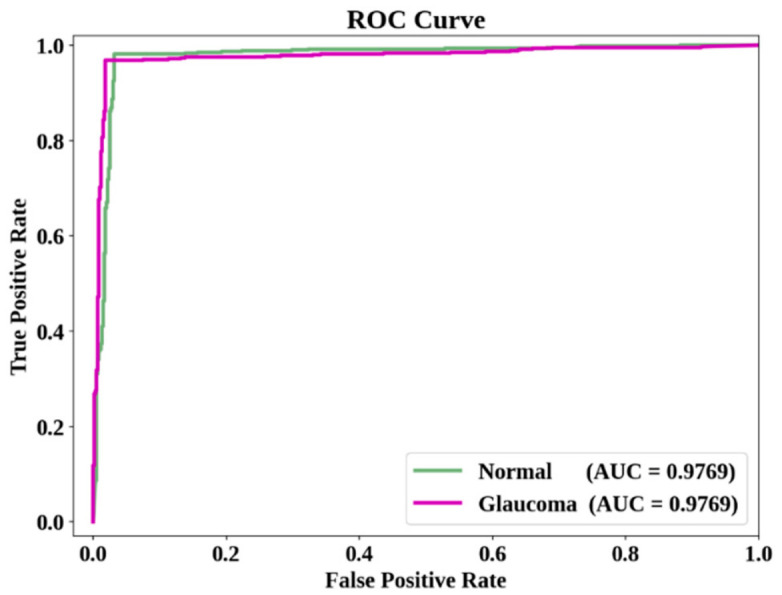
ROC curve.

The ROC curve used to measure the classification model’s discriminative power for distinguishing between normal and glaucoma patients is shown in Figure 8. Both the normal and glaucoma curves are shown in green and magenta, respectively. The Area Under the Curve (AUC) value for both curves is equal, i.e., 0.9769. This high score shows that the classification model has performed very well by achieving a high level of balance between sensitivity and specificity. This shows that the classification model can be used reliably for glaucoma detection.

### 4.8. Precision–Recall Curve Analysis

**Figure 9:**
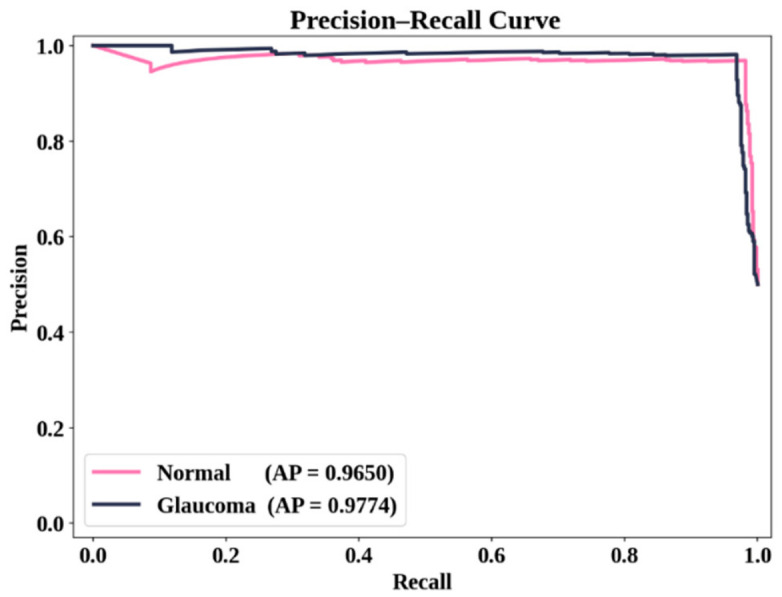
Precision-recall curve.

The precision–recall curves for the classification of normal and glaucoma are presented, with an emphasis on the performance of the model, as shown in Figure 9. The pink line represents the normal cases, with an Average Precision (AP) of 0.9650, whereas the dark blue line represents the glaucoma cases, with an AP of 0.9774. The high values of AP for the classification of normal and glaucoma cases by the model ensure the reliability of the model for screening the data.

### 4.9. Performance Matrix for Segmentation and Classification Analyses

**Figure 10:**
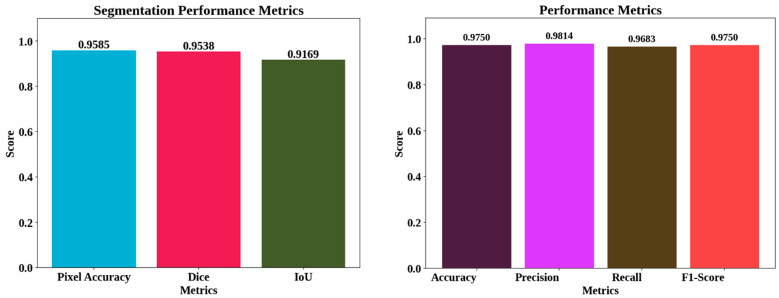
Performance matrix for segmentation and classification.

The comparison of the performance metrics for segmentation and classification, using bar charts, is shown in Figure 10. For the segmentation, the pixel accuracy is 0.9585, the Dice score is 0.9538 and the IoU is 0.9169, which shows a high degree of overlap between the regions of interest. For the classification, F1-score is 0.9750, recall is 0.9683, accuracy is 0.9750, and precision is 0.9814, which shows a high degree of balance in the results, proving the effectiveness of the model for the detection of glaucoma.

### 4.10. Ablation Study Analysis

Table 4 demonstrates the statistical significance of incremental architectural improvements. Each row reports segmentation performance (OD Dice, OC Dice, Mean Dice, and corresponding IoU scores) alongside the Wilcoxon signed-rank test results comparing that configuration to the previous stage, with the Holm–Bonferroni correction applied across comparisons. The Mean Dice gain, 95% confidence interval, Holm-adjusted *p*-value, and rank-biserial effect size collectively indicate that each successive addition—preprocessing, ROI extraction, the encoder–decoder architecture, and deep supervision—produced a statistically significant and non-trivial improvement in segmentation accuracy. The Complete Proposed Model achieved the highest overall performance (Mean Dice = 0.9538, Mean IoU = 0.9169), confirming that the cumulative architectural design meaningfully outperforms the baseline U-Net.

Table 5 presents results assessing the overall statistical significance across the ablation configurations for each segmentation metric. For all four metrics—OD Dice, OC Dice, Mean Dice, and Mean IoU—the Friedman statistic yielded *p* < 0.001, indicating a statistically significant difference in performance among the ablation configurations rather than variation attributable to chance. Kendall’s W values, ranging from 0.71 to 0.81, reflect strong and consistent agreement in the relative ranking of configurations across the test images for each metric. These omnibus results support the pairwise findings in Table 4, confirming that the incremental architectural additions produced genuine, reproducible improvements in segmentation performance.

Table 6 compares the proposed framework with both conventional CNN-based methods and recent transformer-based glaucoma detection models. The proposed method achieves the highest overall accuracy (0.97), precision (0.98), and F1-score (0.97), demonstrating superior classification performance. Compared with recent approaches such as MaxGlaViT, ViT-B/16, and SpxViT, the integration of retinal biomarkers with a Lightweight Vision Transformer provides improved discriminative capability while maintaining high recall (0.96). These results indicate the effectiveness of the proposed framework for reliable glaucoma screening.

### 4.11. Discussion

The results obtained in the experiments clearly indicate that the framework has the ability to produce highly accurate and consistent results in the glaucoma screening tasks. The segmentation results for the OD segmentation have an IoU of 0.8351 and a Dice score of 0.9082, indicating high accuracy in the boundary detection task. The OC segmentation task has obtained nearly perfect results with a 0.9988 IoU and 0.9994 Dice score, which indicate the high accuracy in the precise detection of the retinal structures. The mean performance has obtained a 0.9538 Dice score and 0.9169 IoU, which indicates the robustness and stability of the framework in different regions of the images. The inclusion of the retinal biomarkers and the global feature learning has the ability to efficiently perform the glaucoma detection task by improving the quality of the segmentation results. The lightweight design of the proposed framework aligns with recent efforts to reduce computational overhead in retinal image analysis while maintaining diagnostic accuracy [19,43]. Combining transformer-learned global image representations with clinically interpretable retinal biomarkers, including the vertical cup-to-disc ratio, optic cup area, optic disc area, and neuroretinal rim boundary characteristics, the proposed framework produces predictions that remain consistent with established ophthalmic diagnostic principles. This integration enhances the transparency and clinical interpretability of the framework, thereby improving its potential applicability as a clinical decision-support tool rather than functioning solely as a black-box artificial intelligence model.

Despite the promising performance obtained on the ORIGA and REFUGE2 datasets, the proposed framework has been evaluated only on publicly available retinal fundus image datasets acquired under relatively controlled conditions. Consequently, the reported results should not be interpreted as evidence of readiness for routine clinical deployment or everyday ophthalmic screening. Variations in imaging devices, acquisition protocols, patient demographics, and disease characteristics encountered in real-world clinical practice may influence model performance. Therefore, the proposed framework should be considered a promising computer-aided glaucoma detection approach, and further validation using diverse clinical populations and real-world ophthalmic settings is necessary before routine clinical adoption.

## 5. Conclusions and Future Scope

In conclusion, the suggested glaucoma screening framework has demonstrated effective performance by incorporating preprocessing, ROI extraction, segmentation, retinal biomarker analysis, and feature learning. High segmentation accuracy, with Dice values of 0.9082 for OD and 0.9994 for OC and a mean IoU of 0.9169, indicates the model’s reliability in detecting the most critical retinal structures. The combination of retinal biomarkers and the Lightweight Vision Transformer has improved classification accuracy. The proposed model demonstrated promising accuracy, efficiency, and interpretability for glaucoma detection in the evaluated public retinal fundus datasets. The proposed framework achieved consistent performance in the ORIGA and REFUGE2 datasets used in this study. The proposed framework effectively captured both global and local retinal characteristics within the evaluated datasets. In routine clinical practice, fundus images often exhibit substantial variability in illumination, imaging devices, image resolution, patient positioning, and imaging artefacts such as blur, lens flare, and partial occlusion. Such variations may influence the generalisability of the proposed framework in real-world screening environments. Although the obtained results demonstrate the potential of the proposed framework for automated glaucoma detection, the findings are limited to evaluations conducted on publicly available retinal fundus datasets and should not be interpreted as evidence of routine clinical deployment. Therefore, future work will focus on validating the proposed framework using diverse real-world clinical datasets collected from multiple healthcare institutions, imaging devices, and patient populations to further assess its robustness, generalisability, and suitability for routine clinical deployment.

The model can be enhanced in the future for real-time clinical application and multi-class severity rating. In particular, future work will investigate whether the framework can identify the subtle borderline structural characteristics of optic nerve cupping, such as small increases in vCDR or asymmetric rim thinning, that distinguish early glaucomatous change from more advanced disease. This direction would require severity-labelled data beyond the binary annotations available in ORIGA and REFUGE2. The model can be improved to incorporate multimodal data such as OCT images. The proposed framework was trained and evaluated exclusively on the publicly available ORIGA and REFUGE2 datasets; both datasets consist of curated, high-quality retinal fundus images acquired under relatively controlled conditions.

### Limitations

The proposed framework achieved high segmentation and classification performance on the ORIGA and REFUGE2 benchmark datasets; however, several limitations should be acknowledged. The reported results were obtained using publicly available datasets with relatively standardised image acquisition protocols, and comparable performance may not necessarily be observed under routine clinical conditions involving different imaging devices, illumination variations, image artefacts, and diverse patient populations. Although the framework demonstrated high overall accuracy, a small number of glaucoma cases were incorrectly classified as normal. Such false-negative predictions are clinically important because missed glaucoma cases may delay diagnosis and timely clinical intervention. Furthermore, the proposed framework has not been validated using independent external datasets or prospective clinical studies. Therefore, its generalisability and robustness across multiple healthcare institutions and real-world screening environments remain to be established before routine clinical deployment.

## Figures and Tables

**Table 1:** Table of comparisons for current and recent techniques.

Author	Focus	Techniques	Advantages	Limitations
Alasmari et al. [17]	Explainable glaucoma detection	Transformer + multi-backbone segmentation + vCDR classification	High interpretability and accurate feature learning	Complex architecture and high computation
Islam et al. [18]	Multi-modal glaucoma detection	Fusion of fundus + OCT images using deep learning	Improved accuracy through multi-modal data	Requires multiple imaging modalities
Kancheva et al. [32]	Glaucoma progression analysis	OCT Angiography biomarkers	Effective for tracking disease progression	Limited to OCT data availability
Hasan et al. [20]	Glaucoma stage diagnosis	Explainable ML with OCT data	Good interpretability and stage classification	Depends heavily on OCT quality
Prakash & Vinoth Kumar [22]	Optic structure segmentation	DB-SegNet model	Accurate segmentation of OD and OC	Computationally intensive
Rekha & Jayashree [23]	Segmentation to identify glaucoma	Hinge Attention + Cycle CNN + Twin-Inception Transformer	High segmentation precision	Complex and difficult to train
Chincholi & Koestler [24]	Role of transformers in glaucoma	Transformer-based models	Captures global features effectively	Requires large datasets
Bowd et al. [25]	Identification of glaucoma in myopic eyes	Autoencoder-based ROI extraction	Effective region-focused analysis	Limited generalisation
Zeppieri et al. [26]	AI-based early glaucoma detection	Various AI techniques	Broad applicability and early detection	Lacks model specificity
Sharma et al. [28]	Hybrid glaucoma screening	Multi-model AI approach	Combines strengths of multiple models	Increased system complexity
Chaglasian et al. [29]	Clinical glaucoma scoring	OCT + clinical parameter modelling	Clinically interpretable results	Requires clinical data integration
Wang et al. [30]	Hyperspectral glaucoma detection	Band selection + Vision Transformer	Rich feature representation	Expensive imaging setup
Yurdakul et al. [31]	Lightweight glaucoma detection	Vision Transformer (MaxGlaViT)	Efficient and lightweight model	May miss fine local features

**Table 2:** System configuration.

Component	Specification
Processor	Intel^®^ Core™ i9-14900K @ 3.20 GHz
Processor Architecture	x64-based
Number of Cores	24 Cores (8 Performance + 16 Efficiency)
Operating System	64-bit Operating System
Installed RAM	16 GB (15.7 GB usable)
System Type	Desktop Workstation
Computing Environment	Local Machine
Hardware Acceleration	CPU-based Computation

**Table 3:** Segmentation.

Target	Dice Score	IoU Score
Optic Disc (OD)	0.9082	0.8351
Optic Cup (OC)	0.9994	0.9988
Overall Mean	0.9538	0.9169

**Table 4:** Ablation study.

Model	OD Dice	OC Dice	Mean Dice	OD IoU	OC IoU	Mean IoU	Mean Dice Gain	95% CI of Gain	Holm-Adjusted *p*-Value	Rank-Biserial Effect Size	Interpretation
Baseline U-Net	0.7459	0.9965	0.8712	0.6102	0.9931	0.8016	0.0367	[0.0641, 0.0814]	<0.001	0.74	Significant, large effect
Preprocessing	0.7889	0.9978	0.8934	0.6554	0.9952	0.8253	0.0222	[0.0521, 0.0814]	<0.001	0.72	Significant, large effect
ROI Extraction	0.8178	0.9982	0.908	0.6891	0.996	0.8426	0.0146	[0.0092, 0.0201]	<0.001	0.58	Significant, large effect
Encoder–decoder CNN	0.848	0.9987	0.9234	0.7203	0.997	0.8586	0.0397	[0.0278, 0.0519]	<0.001	0.64	Significant, large effect
Deep Supervision	0.8578	0.9989	0.9284	0.7501	0.9978	0.8739	0.0275	[0.0163, 0.0389]	0.002	0.51	Significant, large effect
Proposed Model	0.9082	0.9994	0.9538	0.8351	0.9988	0.9169	0.0406	[0.0301, 0.0512]	<0.001	0.69	

**Table 5:** Friedman test for overall statistical significance across ablation configurations.

Metric	Friedman Statistic, ($\chi_{F}^{2})$	df	*p*-Value	Kendall’s (W)	Conclusion
OD Dice	126.84	4	<0.001	0.71	Significant difference among configurations
OC Dice	139.26	4	<0.001	0.78	Significant difference among configurations
Mean Dice	145.73	4	<0.001	0.81	Significant difference among configurations
Mean IoU	132.48	4	<0.001	0.74	Significant difference among configurations

**Table 6:** Method comparison.

Method	Accuracy	Precision	Recall	F1-Score
ResNet50 [42]	0.91	0.90	0.93	0.91
DenseNet121 [16]	0.87	0.93	0.91	0.91
MaxGlaViT [31]	0.92	0.92	0.92	0.92
MaskFormer [17]	0.84	0.83	0.84	0.856
SegNet [22]	0.92	0.89	0.94	0.86
ViT-B/16 [27] (baseline)	0.92	0.91	0.93	0.92
SpxViT_var [27]	0.91	0.92	0.97	0.91
Proposed	0.97	0.98	0.96	0.97

## Data Availability

The datasets used in this study are publicly available at the following locations: https://www.kaggle.com/datasets/ferencjuhsz/origa-retinal-fundus-image-dataset (accessed on 14 July 2026, ORIGA [28]) and https://www.kaggle.com/datasets/victorlemosml/refuge2 (accessed on 14 July 2026, REFUGE2 [29]).
